# Problem Mechanism and Solution Strategy of Rural Children’s Community Inclusion—The Role of Peer Environment and Parental Community Participation

**DOI:** 10.3389/fpsyg.2021.772362

**Published:** 2022-01-24

**Authors:** Ying Xu, Ligang Wang, Wanyi Yang, Yi Cai, Wenbin Gao, Ting Tao, Chunlei Fan

**Affiliations:** ^1^CAS Key Laboratory of Mental Health, Institute of Psychology, Beijing, China; ^2^Department of Psychology, University of Chinese Academy of Sciences, Beijing, China

**Keywords:** community inclusion, psychological developmental environment, rural children, traditional group play, mobile phone use

## Abstract

Early childhood development intervention has gained considerable achievements in eliminating intergenerational transmission of poverty in rural areas. Paying further attention to rural children’s community inclusion can also promote the sustainable development of the village. However, there is a lack of systematic theoretical constructs on the village inclusion of rural children. In this study, an attempt was made to explore the problem mechanism and solution strategy of community inclusion of rural children using a grounded theory approach of in-depth interviews. Seventeen parents of children in a national-level poverty-stricken county in Inner Mongolia of China were investigated, adopting the strategy of intensity sampling. The results revealed that (1) the content of rural children’s activities demonstrates enhanced participation in the virtual environment and weakened participation in the real community environment. That is, the activities are characterized by more virtualization and individualization. (2) Rural parents and community peers are two major channels for children’s community inclusion, while both the community peer environment and parental community participation show a weakening trend. This may be an important reason for the virtualization and individualization of the children’s psychological development environment. (3) Developmental intervention programs for rural children in poverty-stricken areas should focus on the reconstruction of children’s community peer environment, encourage the community participation of parents, and fully mobilize local-based educational resources.

## Introduction

Relying solely on economic growth cannot promote equitable and sustainable development in rural and poverty-stricken areas. Only by investing in human development and increasing access to public services can we maximize the revitalization of the countryside and improve social cohesion. Fu et al.’s (2020) qualitative research on the endogenous motivation mechanism of poverty alleviation revealed the psychological mechanism of generations of poverty. They pointed out that substantial companionship and nurturing are important family social capital for the children of poor families to get rid of poverty. Heckman (2000) also suggested that investing in children’s capacity development is a kind of pre-distribution, which is more efficient and fairer than redistribution. The investment with the greatest social return is for disadvantaged children living in a poor family nurturing environment.

Early Childhood Development (ECD) refers to the comprehensive development of early childhood physical, cognitive, emotional, social adaptation, and language (UNICEF, 2015). Western countries have been practicing for a long time to solve the generational transmission of poverty by promoting the early development of poor children, such as the Head Start (Garces et al., 2002) in the United States and the Sure Start in the United Kingdom (Glass, 1999). Recent research and practice also showed that early childhood development interventions can improve the later benefits for executive function, emotional and behavioral health, school preparation, academic achievement, and social participation (Watts et al., 2018; McCoy et al., 2019; Bierman et al., 2021). Besides, the ecology of childhood suggested that socio-environmental factors (e.g., family, community, institutional, and service settings) are the main determinants of children’s wellbeing and psychosocial outcomes (Hertzman, 2010). Neighborhoods, in which children live and grow, influence children’s developmental outcomes (Goldfeld et al., 2015). Furthermore, the developmental niche theory (Super and Harkness, 1986, 1997) illustrated a more relevant point of view. It is suggested that the physical and social settings, customs and practices of child care and rearing, and the psychology of the caregivers are three interactive subsystems of parent-child joint engagement in children’s daily lives, which support children’s play and learning. More importantly, this developmental niche with three components can constantly adjust against the influence of outside sociocultural change (e.g., changes of the original hometown culture) and restore internal stability (Lin et al., 2019). This function provides extensive conditions for full and flexible intervention and long-term construction of a child development environment. Therefore, it is essential and feasible to intervene in the early childhood development environment to promote the quality of human capital in poor rural areas, even get rid of poverty and achieve long-run development.

Current early development promotion projects for poverty-stricken children in China mainly draw on the practices of Western developed countries in terms of content and models and have achieved certain results (Luo et al., 2019; Yue et al., 2019). However, while drawing lessons from Western experience, we cannot ignore the difference between the spatial distribution of poor children in China and developed Western countries. In most developed countries, poor children are mostly concentrated in poor communities in cities, while more poor children in China are in rural areas. Therefore, in the implementation of the early development program for poor children, it is necessary to consider the dual cultural structure of Chinese urban and rural areas and build child development promotion projects based on local culture.

At present, with the development of social economy and rural urbanization in China, the labor force of the rural population is gradually moving to cities, which has an impact on the structure and environment of traditional rural communities (Hu et al., 2014; Yang, 2020). The original acquaintance society in rural areas has transformed into a semi-unfamiliar society, and interpersonal connections in communities have become increasingly alienated. In addition, the popularization of mobile Internet and social media has expanded the virtual environment in the development of children, and rural children are facing the disappearance of traditional game communities (Liu and Shen, 2012). All these conditions make the social and emotional connection between rural children and the community insufficient, so rural children may have problems integrating into rural communities.

Rural children are the future successors of rural construction and development. Children’s inclusion into rural communities can enhance their sense of identity and belonging to the village, and deepen the collective memory of the village (Wu, 2019). The deep emotional connection between children and the village helps encourage them to build their hometown after receiving a good education in the future. In addition, the creation of a good community education environment is the key to giving children substantial companionship and care and promoting the comprehensive development of early childhood. For example, it is worthy to pay significant attention to establishing an interpersonal network between caregivers to promote experience sharing in the local community, which in turn creates opportunities for children to communicate with their peers and promote the development of their language and social skills. What’s more, based on the family or community, people with in-depth knowledge of nutrition, health, and motivation will provide training and interaction for mothers and other caregivers. This community building for the early childhood development environment is one of the most cost-effective investment strategies. It helps break the intergenerational transmission of poverty and increase rural productivity and social cohesion over a longer period of time. Above all, it is particularly important to build a good community inclusion environment for rural children.

However, existing research on community inclusion mainly focuses on the living situation and community inclusion work of the floating population (Duquette-Rury, 2016; Mohammadi, 2019; Yi and Liang, 2020), the disabled or the mentally ill (Gray et al., 2014; Hiranandani et al., 2014; Slater et al., 2020) and the elderly (Biniok et al., 2016; Ronzi et al., 2018). The exploration and interpretation of impact mechanism and intervention measures of rural children’s community inclusion are still limited. Few studies examined the theoretical system of community inclusion of rural children based on the Chinese local situation.

Therefore, the aim of this study is to explore the factors and intervention mechanisms of rural children’s community inclusion. With a view to improving the environment for early childhood development, exploring county-level psychological poverty alleviation models, building a rural social- psychological service system, and better promoting the revitalization and development of poverty-stricken areas.

## Literature Review

### Community Inclusion

“Each place has its own way of supporting its own inhabitants.” This proverb reflects the relationship between individual development and the hometown. Each region has its own unique soil and water environment and social environment. People with natural attributes interact materially with the soil and water environment, while people with social attributes interact spiritually with the social environment through social inclusion. More importantly, social attributes with spiritual interaction are intrinsic attributes of humans, requiring that the individual must integrate into the current social environment to achieve the development of individuals and groups. In other words, based on common emotions, ethics, beliefs, or values, social inclusion as a social bond, links the relationships between individuals and individuals, individuals and groups, and groups and groups with the characteristics of combination or attraction (Chen and Sun, 2012). Community inclusion is specific social inclusion in the community environment. Individuals are integrated members of the community, enjoying needed support from others, and participating in normal community activities with other people (Shalock and Verdugo, 2002).

Based on the perspective of social equity, social inclusion is the process by which individuals gain mutual access to each other’s memories, emotions, and attitudes by sharing their histories and experiences with other members of the community and ultimately integrating them into a shared cultural life (Chen and Sun, 2012). Village inclusion is the concretization of farmers’ social inclusion in the village. Specifically, the village is a “society of acquaintances,” based on blood relations and geopolitical relations. Farmers join this “society of acquaintances” through practical activities with their neighbors, forming a sense of belonging and identity to the village and finally realizing village inclusion.

Based on the perspective of citizenship, foreign scholars chiefly highlight the social inclusion of immigrants, while Chinese scholars focus on the urban inclusion of migrant worker groups and their accompanying children. Existing studies on the social inclusion of immigrants and migrant workers mainly concenter on their economic, social, psychological, or cultural inclusion after leaving their birthplace and entering the city. For instance, in Berry’s psychological-cultural adaptation model, he pointed out that individuals in cross-cultural social inclusion identify with either the original culture or the urban culture, or both, or neither, which corresponds to four cultural adaptation strategies: separation, assimilation, integration, and marginalization (Berry, 1994). Separation and marginalization strategies may bring about severe adverse effects on self-esteem and other psychological outcomes. Zhang et al.’s (2018) study also supports this perspective, which found a significant relationship to exist between dual identity and psychological adjustment. High dual identifiers had better life satisfaction and lower depressive symptoms, emotional loneliness, and social loneliness. Therefore, whether rural children want to integrate into urban life or stay in their hometown in the future, it is of positive significance for children’s lives to form an identity with the original culture of the village.

### Urbanization and Community Inclusion

Urbanization is an important driving force for rural modernization and economic development. However, the in-depth development of urbanization will inevitably bring about a series of social contradictions and problems. At present, with the continuous acceleration of the urbanization process in China, a large number of rural populations, especially young and middle-aged populations, are gradually leaving the countryside, leading to the loss of human resources in rural areas (Yang, 2020). The living space of farmers and their children is changing drastically, exacerbating houses vacancy farmland, and infrastructure abandonment. The phenomenon of “hollowing out” and “decentralization” in rural areas has intensified, and the country is evolving from a “society of acquaintances” to a “society of semi-strangers.” Problems such as the aging population and “left-behind” problems have caused a series of social conflicts and personal problems in rural areas (Xie et al., 2014; Zhong et al., 2017; Thapa et al., 2018). Especially for rural children, due to the limited economic, time, and ability of their parents or grandparents, guardianship over left-behind children are often absent. However, the early comprehensive development of childhood cannot separable from the participation of parents. The inadequate family supervision and emotional care may lead to the weakening of rural children’s family functions in education, emotion, protection, and management, which in turn affects left-behind children’s academic performance, mental health, ideological and moral cultivation, and self-identity (Li, 2015; Sun et al., 2015; Wang X. et al., 2015; Tang et al., 2018).

In this context, some rural children move to cities with their parents and are collectively known as migrant children; some children stay in the country, live with other relatives, and are collectively called left-behind children; and other children, whose parents are both farming in their hometown, are considered rural children in the traditional sense. In terms of children’s community inclusion, part of the current research in China focuses on the inclusion of migrant children in urban communities, while the other part studies the community inclusion of children who still live in rural areas (including left-behind children and non-left-behind children). For example, Li’s (2015) study analyzed the social inclusion of rural left-behind children in three levels of dimensions, social adaptation, self-identity, and acceptance by other groups, revealing the dilemmas of left-behind children in social inclusion. In addition, Lv and Yu (2016) pointed out that the relative backwardness of the rural economy is the root cause of the cultural identity crisis, while the absence of rural culture in education and the cognition and behavior of family fathers who aspire to the city also has an undesirable impact on children. Furthermore, Wu (2019) analyzed the five dimensions of satisfaction, village living environment, interpersonal relationships, community participation, emotional attachment, and inheritance tendency, and found that children in Jiangwan Village have a low level of hometown identity.

### Peer Environments and Community Inclusion

In traditional agricultural or tribal societies, two age groups generally exist adults and children. Ecological research revealed that the coming-of-age tradition was the dividing line for the transformation of individual child identity into adult identity. The specific developmental mechanism was that the coming-of-age ceremony helped children be liberated from their original family and integrated into the adult group of the village. However, Harris’s (2015) theory of group socialization went further by stating that children left the activity range of their family at age three and then join their peer playgroup, rather than adult groups. The coming-of-age ceremony just serves to bring them and other peer partners into a new social category, in which they are expected to assume the duties and responsibilities of adults (Harris, 2015). As a result, children gradually realized the transformation of their social identity. Because socialization can contribute to the realization of identity changes in group games, and it is also a change process in children’s cognition and social patterns. Consistent with this notion, many studies indicated that peer play or group play-based activity is a powerful vehicle to promote learning, development, socioemotional processes and social interaction skills (Fung and Cheng, 2017; Tunçgenç and Cohen, 2018), especially for low-income and otherwise disadvantaged children (Nicolopoulou et al., 2015). Harris also emphasized that children’s peer relationships play an essential role in integrating them into their communities. In addition, prior studies suggested that the community can provide a place for young children to play together and accompany each other by setting up game activity rooms and reading rooms. These settings have expanded the range of community activities for children and their families, improved children’s sense of belonging and membership to the community, positive social relationships and learning potential (Park et al., 2021). In line with this view, groups of children also play a vital role in traditional rural life in China, specifically, older children taking care of younger children, children playing traditional group games with their peers, children visiting each other’s homes with other peers, and so on. These life experiences form the collective village memory and promote children’s village inclusion and original cultural identity. However, with the urbanization of rural areas and the expansion of the virtual environments, the traditional group play environment in the community is gradually disappearing. Such an unconnected community environment cannot meet the children’s needs for peer companionship and social interaction in the early development process, which is not conducive to children’s emotions, academic success, self-identity and social development. Nonetheless, it is still feasible to recover traditional collective games, which can be combined with community construction. Neighborhood services cover a range of elements, including access to parks and playgrounds, street lighting, footpaths, community activity rooms, which basic services can be combined with children’s group games to create conditions for the recovery of traditional group games (Goldfeld et al., 2015).

### Virtual Environments and Community Inclusion

Johnson and Puplampu (2008) proposed the ecological technology microsystem theory, which is a development and supplement to ecosystem theory in the context of the rapid development of electronic technology. The theory systematically discussed the positive and negative effects of electronic media use on children’s cognitive development and social behavior. Moreover, the same electronic media use behavior has different influences on different people. For example, some studies have found that online activities can satisfy users’ basic psychological needs, such as a sense of autonomy, a sense of belonging, and a sense of accomplishment. However, there is an effect of the rich getting richer and the poor getting poorer. Specifically, for those who have their basic psychological needs met in real life, the satisfaction obtained from online activities can enhance their well-being. In contrast, those who cannot satisfy their basic psychological needs in real life will not improve their well-being despite the satisfaction of their psychological needs being perceived online (Wang L. et al., 2015).

In a community environment, if mobile and digital technologies can be used to develop a virtual community platform that is conducive to the health, education and daily life of community residents, then this virtual environment will help the community to integrate. For example, research shows that virtual community inclusion with using mobile health and digital technologies in mental health was positively associated with positive emotions and significantly predicted recovery (Shpigelman et al., 2021). However, if parents or children have problematic mobile phone use, the communication between parents and children will be neglected. The serious lack of family education and care may bring about severe adverse effects on children’s early development. In addition, there will also be a lack of contact between neighbors, which is likely to form a gap between community residents. The collective consciousness of residents will be constantly weakening, affecting the stable and harmonious environment in the countryside (Yang, 2020). Related research shows that phubbing has become a common phenomenon in family life. Family members would be distracted by a mobile phone while in the company with each other. Specifically for individuals with a low level of self-control, parental phubbing and adolescent problematic mobile phone use would have more negative effects on the parent-child relationship (Niu et al., 2020).

Therefore, in keeping with grounded theory, this study will explore the problematic mechanisms and possible solution strategy of rural children’s community inclusion. On the one hand, it is necessary to objectively present the ideas and attitudes of rural parents and the reality of rural children’s development. On the other hand, we aim to thoroughly explore educational resources based on local culture, together with rural parents, to enhance the operability of future intervention programs.

## Research Design

### Research Method

The village inclusion of rural children is a complex and dynamic process. It is necessary to objectively present the views and micro-interaction behaviors of parents and children, as well as a multidimensional rural children development environment. Qualitative research methods are more feasible to comprehensively study these matters of concern. Therefore, this study utilized in-depth interviews and grounded theory (GT) to collect and analyze the data. Grounded theory is an important qualitative research method that allows for elucidating the overall defined pattern of the complex phenomenon and discovering theoretical frameworks from rigorous data collection and analysis (Glaser et al., 1968). As a sociological method, grounded theory has been widely employed in the fields of community education, community engagement, and social work research (Charmaz, 2006; Hastings et al., 2011; Claramita et al., 2019), which also proves that grounded theory has good support for studying community inclusion in this study.

### Sampling and Participants Characteristics

The interviewees in this study are mainly from two villages (Village A and Village B) in a poverty-stricken county in Inner Mongolia. The main source of income for farmers in the two villages is animal husbandry in the farming area, with about 10 mu of arable land. The two villages are both Mongolian and Han mixed living patterns, and Mongolian residents can also communicate in Chinese. Village A has a population of 732 people, of which 46% are Mongolians and 13% are migrant workers. The per capita annual income is 12,800. Village B has a population of 271 people. Mongolians account for 55%, and migrant workers account for 16%. The per capita annual income is 14,500. The living spaces of the two villages consist of village houses, cattle and sheep pens, activity squares, villagers’ activity rooms, prairie book houses and vast fields outside the village. In the early stage, our project team utilized the villager activity room and the prairie book house to carry out community construction work. A special children’s activity room has been established to encourage rural parents to jointly optimize the environment for the development of children’s mental health. In this context, this study discussed with the interviewed parents the implementation of parenting mutual support groups to improve the development environment of rural children.

Based on the principles of objective sampling and theoretical sampling, this study applied maximum variation sampling and snowball sampling to select typical cases with large information intensity and variation. The selection of the sample size is based on the principle of data saturation. When the interview data reaches a stable consistency between categories, the variation and saturation of theoretical phenomena, and the depth of focus, the interview was stopped (Fu et al., 2020). A total of 15 child guardians from two villages were selected as interviewees, with ages ranging from 24 to 48 (seven from 20 to 30, seven from 31 to 40, and one from 41 to 50). Among the 15 interviewees, one was the child’s maternal grandmother (parents working outside the home), and the remaining 14 were the child’s mother. Their education degrees ranged from elementary to undergraduate (one in elementary school, seven in middle school, five in high school, and two in undergraduate). There were 13 farmers and two staff members in public institutions. Moreover, 6 mothers had children aged 0-6 as well as children in elementary school. More importantly, in the process of analyzing the data, we added two guardians from urban families to compare the mobile phone usage of rural and urban children. These two guardians are both child’s mothers, working in public institutions. The first mother is 35 years old with high school education. She has a 5-year-old son and a 2-year-old daughter. Another mother is 34 years old, bachelor’s degree, and has a 3-year-old son. Above all, there are a total of 17 interviewees in this study.

### Collection of Qualitative Research Data

To collect comprehensive and effective information, we selected face-to-face interviews and semistructured interviews for this research.

#### Pre-interview

Before the in-depth interviews, two guardians of rural children were selected as interviewees to conduct the pre-interviews, which provided the basis for the formal interview outline design. The pre-interviews mainly focused on the core theme of the “child development environment.” In addition, the guardians were invited to answer freely according to their own ideas, reporting on the way their children play, their expectations for the children’s future development, and current problems existing in the children’s development. The preliminary findings showed that (1) all the farmers in the sampling area could communicate in Mandarin, so it was convenient to transfer the interview scripts into the text; (2) rural children’s guardians had their own opinions and thoughts on the topic of the interview, which ensured that the interviews could obtain the needed information; and (3) regarding the core issue “child development environment,” although the guardians also were involved in the children’s growth process, they were more observers, not direct experiencers. Therefore, there was relatively little experiential information provided when parents discussed the way children play. It was suggested that, in the formal interview, the interviewees needed to offer corresponding memories of their childhood.

#### Formal Interview

According to the data organized from the pre-interview, the final outline of the interview is as follows. (1) Recall your childhood games and talk about the effect of these games on children’s development. (2) Have traditional group games disappeared, why? (3) What is your attitude toward the recovery of traditional group games? How can they be recovered? (4) What is your attitude toward parenting mutual support groups? Would you like to participate? Why? (5) What is your expectation of your child’s future career?

To encourage the interviewees to better enter into the topic situation, the interview for question (1) and question (2) used picture priming. Question (1) presented the interviewees with a series of pictures of traditional group games (e.g., throwing sandbags, hopscotch, flipping rope, bouncing glass balls) to help them recall childhood games. Question (5) showed three pictures of work scenes: an office work scene, a factory workshop work scene, and a farmland farming scene, facilitating interviewees to think about their career expectations for their children. In view of question (4), this interview utilized the new children’s activity room, built by our project team in their villages, as the priming materials to inquire about their attitudes and views on parenting mutual support groups.

The choice of time and place for the formal interview was mainly based on the interviewees’ convenience. When the interviewees had a large amount of free time and were willing to cooperate, the interview was conducted at interviewees’ homes in a quiet and undisturbed environment. When the interview information reached or basically reached saturation, both parties mutually decided to end the interview. In this study, the average duration of 15 interviews was 30.13 min. After the interview, the interviewees were appreciated with gifts. The researchers converted the audio recordings of the interviews into transcripts.

### Data Processing

The researchers used Atlas. ti 8.0 qualitative analysis software to code and count the imported Chinese interview data. The software can index, search, and theorize these non-numerical and unstructured data. Before coding with the software, researchers must first set aside the existing value judgment criteria and just abstract or conceptualize the text content, ensuring that the minimum meaning unit is a reflection of the interviewees’ views or ideas.

## Research Data Analysis

As mentioned above, this study mainly used theoretical coding based on grounded theory to analyze the collected data (Glaser et al., 1968). The analysis procedures include open coding, axial coding (secondary coding), and selective coding (tertiary coding).

During the data analysis, the researcher had to compare and reflect on the whole process of the study at all times. For example, in analyzing the data, we learned that many rural children had the problem of excessive mobile phone use. If comparing this phenomenon with urban families, we may get a more comprehensive understanding of mobile phone use of rural children. Therefore, in the subsequent sampling, two guardians of urban families were supplied to fill the information gap. The codes, categories, and theories in each step were taken into account in all aspects of the case and data. Before opening the coding, the coders listened carefully to the interview recording two or three times to make sure they had a good grasp of the tone and emotional state behind the text message. In the process of coding, the coders always insisted on combining the interview recording and notes to give meaning to the text.

### Open Coding

First, we set the corresponding number for each interviewee according to the order of the interview (e.g., N1, N2……C14, C15, where the numbers represented the order of interviews, N for rural areas and C for cities and towns) and ensured that the source of the code and the citation link were correct. Second, we carefully read the contents of every word, sentence, and paragraph in the imported original materials. With “Child Development Environment” as the core, we searched for repeated meaning units from the materials and extracted the login code numbers of text materials that were meaningful to the research problems. Finally, this study established a total of 73 code numbers, involving 235 node samples.

### Axial Coding

Based on the initial coding system, this study further extracted the meaning reflected by the coding system around “children’s psychological development environment.” The extraction process was based on a continuous comparison method, in which the original coded material was repeatedly compared and refined and divided into different categories. Subsequently, the authors continued to compare repeatedly in the process of classification to maximize the differences among the different genres. The main comparison methods are homogeneous comparison, heterogeneous comparison, horizontal comparison, and vertical comparison. Finally, 11 secondary codes were extracted, among which five open codes (involving 10 node samples), such as “ignorance of white-collar work,” “demand for children’s self-monitoring ability,” “learning supervision,” “natural games,” and “doing what they like,” were not included in the secondary code. The specific open coding and categorization are shown in Supplementary Table 1.

### Selective Coding

This study adopted the coding paradigm of “condition-action-consequence” for selective coding (Corbin and Strauss, 2014). The materials of qualitative research usually contain many complicated concepts and relationships. Paradigm can help researchers extract contextual factors from research data and connect context with the process, as an analysis tool, to deepen the understanding of materials. The basic elements of the paradigm include conditions, actions, and consensuses. Therefore, we utilized the guidance of “condition-action-consequence” to search the information central to the “child development environment” and divided them into appropriate categories. Then, all categories were established connections around the core category and we gradually sorted out the story framework described by the guardians of rural children. The specific content is shown in Table 1.

**TABLE 1:** Main categories and subcategories developed from axial coding to selective coding.

Main category	Subcategory	Number of node samples
Virtual environment	External factors of virtual environment participation	22
	Internal factors of virtual environment participation	3
	Virtual environment participation	30
Professional identity of parent	Low professional identity of parent	26
Community participation of parent	Low sense of community of parent	17
Peer environment	Weakening of peer environment	31
	The function of companion game	28
Peer environment reconstruction	The attitude of peer environment reconstruction	19
	Obstacles to parenting support groups	25
	Conditions of parenting support groups	14
	Benefits of parenting support groups	10

### Validity Test

Validity testing is an essential method to assess the quality of qualitative research. In this study, the validity test was mainly conducted by participant tests and expert evaluation methods.

#### Participant Testing and Expert Evaluation

To avoid the existence of the researcher’s subjective assumptions in the study findings, the researcher fed back the coding and conclusions to the interviewees after arriving at the preliminary results of the study. These materials were tested in terms of both descriptive validity and explanatory validity. The researcher selected three participants, with different education levels and regions, among the interviewees to give feedback on the coding and results. One participant was a parent from a rural area with high school education, and the other two were parents from a county area with a bachelor’s degree.

In addition to the participants in the study, on the one hand, two university lecturers with a Ph.D. in psychology were invited to give feedback on the coding as well as the results, with both testers having rural life experience. On the other hand, one university professor with a Ph.D. in psychology but no rural life experience was invited to conduct the test. Whether the testers had rural life experience was a comparative integration from an inside-outside perspective.

The test and evaluation methods included both quantitative scoring and feedback. Specifically, the quantitative evaluation was scored on a 5-point scale, ranging from 1 (very poor) to 5 (very good). The evaluation results are presented in Table 2. The mean scores of the three tests are above 4, indicating that the validity of the study is good.

**TABLE 2:** Results of evaluative reading assessment.

Type of validity	Test items	Evaluation score						
		Participant 1	Participant 2	Participant 3	Expert 1	Expert 2	Expert 3	Equal split
Descriptive validity	The open code accurately summarized the original words of the cited material	5	5	5	5	5	5	5
	The findings matched the actual situation in the community or village	5	5	5	5	5	5	5
Explanatory validity	The results of the study provided a reasonable explanation for the respondents’ thoughts	5	5	5	4	5	5	4.83

We summarized the feedback from the three experts and addressed it in different ways (see Table 3). The researcher first reflected on the parts of the expert feedback inconsistent with the findings. Then we returned to the original data and examined whether the description and interpretation of the results were well-founded. If there was insufficient evidence, then the original results needed to be revised.

**TABLE 3:** Feedback from experts and the treatment of feedback.

Study results	Expert feedback	Treatment of feedback
Parental professional identity was categorized as community involvement	Farmers’ sense of their identity is generally low, but this may not be the reason for their low community participation. Thus, it is not appropriate to categorize identity as community participation.	Returning to the original data, the analysis revealed that there was indeed insufficient evidence to support a causal relationship, so a tertiary coding for “parental professional identity” was added.
The disappearance of community peer environment for rural children	The information mentions that children get together to play mobile games, which is considered a peer environment, so the term “disappearing” may not be appropriate.	Back to the original data, some parents did mention that children rode bikes together or played on their phones together. Respondents expressed more about the disappearance of traditional group play, therefore, based on which the researcher changed “disappearance of community peer environment” to “weakening of community peer environment”
Playing with toys alone	Does the open coding of “playing with toys alone” reflect a weakened peer environment or external factors of virtual environment participation in the category categorization?	Returning to the original data, it was found that the phenomenon of children playing with toys alone reported by parents was to explain the disappearance of group play. For this reason, the original formulation was retained.
Long hours of homework	Interviews focused on recruiting guardians of infants and toddlers aged 0-6 years, so why did it emerge that parents described their third-grade children?	Six of the mothers interviewed had children in elementary school in addition to children aged 0-6 years. When talking about group play during the interviews, this group of parents also reported on the basis of their older children. Considering that there has been a tradition of older children taking care of younger children in traditional child groups, the definition of community peer environment was not limited to infants and toddlers aged 0-6 years, and the original formulation was retained.
Children community inclusion through peer environments and parents	Are children likely to be directly influenced by the community environment, and should the direct influence of the community environment on children be added to the theoretical constructs?	Back to the original data, we found that one parent reported sometimes working in the field, and he would take his child up the hill with him. Then the child would play with dirt in the field by himself. However, the other parents’ reports did not cover these elements, which may be an information gap to be further added in the future. Therefore, the theoretical constructs of this study remain reserved for children’s community inclusion through parent and peer environments.

#### Avoidance Measures of Other Validity Risks

(1) Validity risks of sampling

Different from quantitative research, sampling in qualitative research puts a greater emphasis on the typicality of the research object rather than the representativeness of the population. This study focuses on the environment of children’s psychological development, which requires a certain reference system to analyze. Horizontally, the developmental environment of urban children can be regarded as a reference system; thus, two parents of the county town were included as samples in this study. Longitudinally, the community environment changes over time. Accordingly, the guardians’ own childhood developmental environment can also play an important role in the reference system. Therefore, when including the samples in this study, parents born in the post-80s and post-90s underwent sample matching. In addition, intergenerational parenting is a typical phenomenon that occurs during the influx of rural labor to urban areas. Therefore, this study selected one case of a family with left-behind children in which the respondent was the child’s maternal grandmother. Such a sampling approach ensures that the study maximizes information density and intensity.

(2) The validity risk of the researcher’s identity as an “outsider”

Although the researcher in qualitative research is mostly an outsider, in qualitative research involving sociocultural phenomena, the researchers first need to reflect on their identities to ensure that they produce theoretical knowledge from the perspective of an “insider” as much as possible. In this case, to better integrate into the primary culture of the interviewees, the interviewers and analysts in this study were all those who had experienced rural life, i.e., their childhood was basically in the country. This not only allows the interview process to follow the research subject’s thinking and achieves in-depth information mining in the interview but also ensures that the collected information is understood from the research subject’s perspective. In addition, to avoid risks to research validity from the researcher’s own childhood experiences, the original words or phrases were retained in open coding as much as possible to prevent personal subjective assumptions.

## Results

### Weakening of Community Peer Environment

The “children group” is an important peer environment for children outside of school, and most parents interviewed expressed positive experiences of playing together in a community peer environment as a child.

*N4: My childhood was simple, with not so many toys. It was fun to play these things with my friends.*

*N11: We didn’t have these toys at all when we were kids. Because these are all made by ourselves. We made or painted all the playthings when we were kids. We jumped and played together. Now the kids don’t have those games, and they can’t play.*

*N12: It feels like a happy time.*

*C14: I think we used to have a much more interesting life than the kids now.*

*C14: Even though there are squares in this neighborhood, people’s kids didn’t know each other that well. Unlike before, when we lived in the hutongs, the relationship was very familiar and then just played so casually.*

Traditional group play is an important way to bring children together and an important purpose for children to gather together. Parents interviewed perceived traditional group plays to be physically active, intellectually developing, enhancing a sense of sharing, improving attention, passing time, promoting a sense of community, enhancing positive emotions, and improving imagination. In particular, these descriptions reveal that children’s group play is a significant means of socializing children in a peer environment. However, almost all parents interviewed reported that these peer environments, in which group play was a bond, had disappeared from the community.

*N4: They rarely play together. My child came back just to find a few children to ride a bike together.*

*N7: It seems that the kids don’t play (these traditional games) now, and my girls don’t play them, either.*

*N9: I wish there were, but it’s likely that no one plays (traditional games) anymore. When we were in school, maybe kids played a little bit more.*

*N11: Now children do not necessarily play these (traditional games). My daughter doesn’t know that these things are played, either.*

*C15: Basically, you can’t see them anymore. Sometimes you may see them kicking a shuttlecock, but the other playthings may not be seen.*

The reasons for the disappearance of group games by the interviewed parents mainly include: (1) the substitution of traditional games by mobile phones, televisions, and new toys; (2) the disappearance of the tradition of older children taking younger children to play; and (3) the weakening of group games and outdoor activities because of many school assignments.

*N4: Three kids sitting together can play for half a day. They each look at their own phone to play the game [Mobile phone use].*

*N13: Yes, the post-80s, post-90s and my mom their post-70s played (traditional games). But the post-00s no longer have access to these games. Because the cell phone is developing too fast [Mobile phone use].*

*N7: Now the child is in the third grade, playing less. Because there is usually a lot of homework even on Saturday and Sunday [More homework].*

*C14: The reason may be that one, is that there are more children now, and the other is that our school environment is in buildings [Outdoor activities are reduced].*

*N6: The key is that the older (children) do not like to play with him [The disappearance of the tradition of older children taking younger children to play].*

The above results suggest that children’s peer environments are moving away from the community and that children are moving away from their peer environments.

### Weakening of Community Participation of Parents

Neighborhood relationships are an important part of the sense of community. Most parents interviewed manifested fewer visits to neighbors, more mobile phone entertainment, and a declining sense of community trust.

*N6: I just don’t have time to take him around. I also worried about delaying others’ work if he goes to neighbors’ homes to play. Otherwise, there are children (playing with him) in the east block [Few visits with neighbors].*

*N9: When other children came to visit, they played for a while and went home. Usually, our kids go outside to play, but there are few children outside [Few visits with neighbors].*

*C14: I think it’s still a problem with the living environment. When I used to live in a hutong, I used to run to any families to play in our community. Which family can we go to play in nowadays? We all don’t talk much to each other [Few visits with neighbors].*

*N4: But once grandparents came back, they both watched* Kuaishou *all day long. When they got older, they each took their phones to watch* Kuaishou *[Parents have more mobile entertainment].*

*N9: But at that time my parents generally did not care about me. I go out to run and play by myself. Nowadays, families do not let children run to play by themselves. Children are followed by adults behind [Community trust decline].*

The above results show that parents’ lifestyle is moving away from the community environment.

### Low Professional Identity of Rural Parents

Parents are the first teachers of their children and an important way for children to identify with their original culture. The rural parents interviewed in this survey all showed low professional identity with farmers. Additionally, they did not want their children to be farmers in the future.

*N1: We are also farmers, what’s the use? Tired and exhausted.*

*N3: That is also very tiring!*

*N4: I think it’s hard to be a farmer. We are farmers too, and we don’t want our children to be like this again.*

*N6: The future of farmers is not yet known.*

*N7: I don’t earn much money, and I’m exhausted.*

*N9: We are farmers. I feel that farmers are quite tired all year round.*

*N10: The income is low and not much help to his future life.*

*N11: Farmers who grow crops are not easy to worry about the amount of rain.*

*N13: This is the least expected. Farmers are very tired to work on the farm.*

The above results indicate that rural parents have a low sense of professional identity and do not want their children to continue to be farmers.

### Virtual Environment Participation of Children

The rural parents interviewed reported that it was common for children to use electronic devices such as mobile phones. Children were also very proficient in how to operate phones. In addition, playing on the phone was an effective way to keep children quiet and not clingy when adults were busy with farming or not able to take care of their children. In terms of content, children mainly watched short videos and cartoons and played some small games.

*N2: She chooses her own. She knows all the operations of the phone and understands it better than her father.*

*N3: Sometimes he doesn’t want to study when I’m working, so I let him play a little.*

*N7: My son uses his cell phone every day now.*

*N9: When (I’m) busy working, she plays on the phone at home by herself.*

*N11: They all look at the phone at home, which may be harmful to children.*

*N12: She only plays Volcano video. How come she is so proficient with Volcano video? She doesn’t play Tik Tok.*

*N13: Yes, we generally sit together and play on the phone, giving him a phone to play for a while to let him stop horseplay.*

Although many parents reported that looking at mobile phones is bad for children’s eyes and health, reports from rural parents interviewed indicate that many children spend a lot of time using mobile phones.

*N5: If you leave him alone, he can play for half a day.*

*N7: My child can watch three or four hours a day.*

*N9: She wants to play, and she can play all day!*

Regarding parents’ behavior of restricting their children’s mobile phone use, the following types can be classified from the interview data: (1) no restrictions; (2) restrictions but no clear standards and rules; and (3) absolute restrictions. It is noteworthy that two parents from the county town reported absolutely restricting their children’s mobile phone use.

*N7: They each had a mobile phone to play on, and then I watched TV, thinking that one person had one phone, avoiding fights. [No restrictions].*

*N8: No discipline for him. Basically, he can look at phones if he wants [No restrictions].*

*N6: Rarely let him look at his phone. Unless when I’m working outside on a cold day and he’s on the bed just waking up, I’ll show him cartoons for a while. In this case, I’m not afraid of him getting cold anyway [Having restrictions but no clear standards or rules].*

*N8: She won’t listen to what I say, yet she listens to her dad. If her dad allows her to watch, she does, and if he doesn’t agree, she doesn’t [Having restrictions but no clear standards or rules].*

*N11: He finished all the homework left by the teacher, and then he wanted to play on the phone. I said that he could watch TV. But TV didn’t seem to appeal to him, either [Having restrictions but no clear standards or rules].*

*C14: We don’t let him use those, including the cell phone. We don’t let him use cell phones [Absolute restriction].*

*C15: We basically don’t have children playing on the phones in our family. We don’t let them use mobile phones [absolute restriction].*

In addition, some parents interviewed reported the phenomenon that parents and children each had a cell phone and used electronic products together. The modeling effect of parents’ mobile phone use habits was regarded as an important trigger for children’s participation in virtual environments.

These results indicate that rural children’s participation in virtual environments is common and lacks supervision in participation duration and interactive content.

### Attitude and Condition of Peer Environment Reconstruction

The results suggest that the psychological development promotion program for rural children should focus on strengthening group intervention and education for rural children and parents from the perspective of community culture. In this way, we can rebuild the community peer environment for rural children and further integrate parents and children into the community environment.

#### Benefits and Obstacles of Peer Environment Reconstruction

The parents interviewed were supportive of rebuilding their children’s peer environment but also had some concerns and obstacles.

Supportive attitudes were mainly reflected in the possible benefits of building parenting support groups, including exercising social skills, strengthening unity, reducing parenting stress, improving parenting efficiency, increasing understanding, enhancing parental responsibility, strengthening cooperation, and enhancing group play.

Concerns and obstacles were mainly reflected in the following areas: worried to trust children to others, fear of strangers for many children, lack of time to take care of children, hard to sustain, fear for others’ children getting hurt, fear of conflicts among children, fear of caregiving responsibilities, and an inability to control multiple children (see the benefits and obstacles of parenting support groups in Supplementary Table 1 for the contents of the source sentences of the respondents).

Although many parents expected the group play of children in the community to be restored, they also said that it was difficult for children to recover naturally. However, it might be possible if parents or teachers could participate in the restoration and reconstruction.

*N4: His aunt (who is a teacher) took the children to play hopscotch once she returned this summer. I saw his aunt with the children drawing like this.*

*N10: That’s okay, too. We took sandbags to school and played with the schoolmates. When we had time after class, we would play turned rope together.*

*N13: I think it will be possible to gather children together to play games.*

#### Conditions of Peer Environment Reconstruction

Through further follow-up interviews on the concerns of the parents interviewed, some of them gave many constructive opinions that could be conditions and strategies for rebuilding the peer environment of rural children.

The first condition for the construction of parenting support groups is parents’ supportive attitudes. A supportive attitude is an important condition for addressing obstacles, such as lack of time to bring up children, insufficient or wrong awareness of early education, and for children to be able to participate in support groups. Without parental support, children cannot choose to participate in group activities in the community on their own.

*N4: The main thing I think is to communicate more with the child’s parents. Now from this clock-in activity (reading picture books), parents are not so active. We need to change parents’ thinking. We need to communicate with parents to improve the awareness that the child can’t be only coaxed not to cry. We also need to guide the children to read more books, which is beneficial. Only when the parent’s awareness is raised, and then they can let more children out to the library to read books.*

Second, mutual trust and setting rules are important conditions to address a range of obstacles, such as worries about trusting children to others, fears that the project will be difficult to sustain, fears of others’ children getting hurt, and fears of caregiving responsibilities. Moreover, other parents interviewed specifically mentioned that it was best not to involve money in this project. This attitude is consistent with the culture of the communities that already exist in rural areas.

*N12: The thing is that people communicate with each other. Familiar people are the same. It’s okay for us to collide with familiar people.*

*N12: Just have a rule in advance. Otherwise, it is very abrupt. That would never work.*

*N13: People are all like this. If he bumped or upset other kids, their parents will definitely think more badly. The key is our relationship. We definitely don’t think much about it, because the relationship is trust.*

Finally, several respondents proposed the demand for professional teacher support. This condition is a significant strategy to solve obstacles, such as the inability to control multiple children, fear of conflicts among children, and fear of strangers for many children.

*N12: The teacher has the authority. We are not teachers. Once there is something (problem), they will certainly hold you accountable.*

*N13: I think parents have no ability to read and talk to them. They can recruit a teacher or somebody to tell them the content.*

*C15: We can ask professional teachers when I have temporary work and no time to come. They can speak to us. I feel that is acceptable.*

### Theoretical Integration

In the final stage of grounded theory research, careful refinement of selective coding can lead or relate to the core category of other categories. This study adopted a bottom-up approach to constructing a theory by iteratively inducting and analyzing original data to gradually generate a theory that can demonstrate a point of view.

**FIGURE 1:**
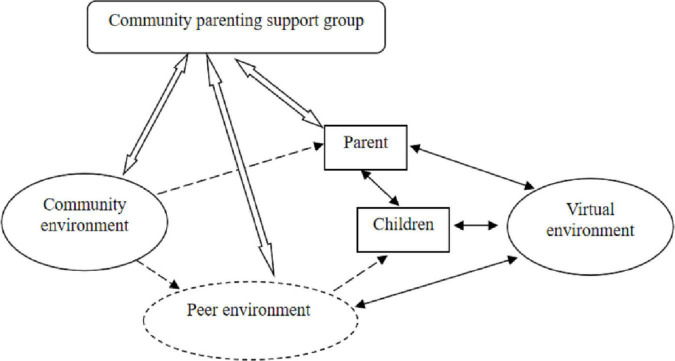
Children’s community inclusion dilemmas and solution strategies. 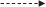 denotes staying away; 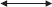 denotes spontaneous interaction; 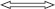 denotes intervention to enhance interaction; 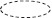 denotes existing environment; 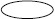 denotes environmental weakening; 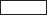 denotes creating environment.

Generally, the theoretical construction of grounded theory research included the following steps. First, it was essential to find the core theme, reflecting the research problem, on the basis of selective coding. In this study, the core theme of “children’s community integration” was developed through the examination and correlation analysis of the established selective coding. Second, the selected core theme was then reverted to the initial material for comparative analysis. Particular attention was given to case materials with opposing or divergent views, which helped to further the theoretical development of the “condition-action-result” linkage of the core themes. Third, the preliminary relationship framework was devolved to the case data to test the compatibility of the theoretical model with the original data. To achieve the best match between the data and the theory, the theoretical model was constantly revised. Finally, there was an examination of the internal consistency of the theoretical system to establish a holistic theoretical perspective statement through the integration of the previous three steps (see Figure 1). This theoretical perspective was based on the “reality” jointly constructed by the researchers and the participants. It could effectively provide reference information for the practice of social intervention.

According to the logical relationship between categories, this study presents a theoretical model for the problem mechanism and solution strategy of rural children’s community inclusion. Specifically, the current rural children’s community inclusion presents the problem of peer environment weakening. The virtual environment is increasingly invading the lives of parents and children. In addition, parents are less involved in community participation. In response to these problems, in the future, community parenting supporting groups can be established to strengthen the construction of community environment, peer environment and parental participation, so as to build a complete community inclusion environment for rural children.

## Discussion

### Virtualization and Personalization of Rural Children’s Activity Contents

This study found that the content of rural children’s activities shows virtualization and individualization. Rural parents may control the use of electronic media for children entering school age, while there are no clear control standards for children’s mobile phone use.

In addition, the results revealed that children’s electronics use behavior is associated with the number of electronics in the home, parental accompaniment and regulation of children’s electronics use, and the level of encouragement of outdoor activities. The two parents from the county had strict restrictions on their children’s mobile phone use, and their children were less likely to participate in the virtual environment. In contrast, rural parents often had no explicit restrictions and even played on phones with their children at home. It is noteworthy that they used their phones in parallel, not accompanying their children to pay joint attention to the phone content. This phenomenon may be an important exogenous factor for children’s participation in the virtual environment. In addition, one parent reported that her child has a strong need to play with other children, but it is difficult for her child to get together with other children to play because of the few or short parental visits. Another parent stated that children currently have good material conditions but actually lack companionship. When companionship needs cannot be fulfilled from the real world, children may seek them from the virtual worlds constructed by electronic media, which is an important internal reason for children’s participation in virtual environments.

### Simplification of the Developmental Environment for Rural Children

The virtual and personal characteristics of the content and format of rural children’s activities are the evaluation conclusions based on both horizontal and vertical reference systems. This study found that it is widespread for rural children to use electronic devices, meaning that electronic devices are becoming prevalent much faster than researchers’ perceptions of the impact of electronic devices on children. At the individual level, it is difficult to give a definitive conclusion regarding whether children should or are appropriate to use electronic media. However, from the view of the developmental environment, it may be more helpful to understand the impact of electronics on children’s development.

First, in a longitudinal comparison, the developmental space for rural children used to consist of the school life environment, the peer group environment, the virtual environment (e.g., television), and the parental companionship environment. In the past, once children became capable of role-playing games, parents were rarely involved in children’s activities. It can be argued that traditional group play has helped children move from home to the community and achieve social inclusion (Luo, 2016). Nevertheless, the preliminary findings indicated that traditional group play has disappeared in rural areas, and children’s community peer environment tends to be weakened. The comparison shows that the current developmental environment of rural children consists of a school life environment (kindergarten), a virtual environment (mobile phone, TV, etc.), and a parental companionship environment. Moreover, children’s access to rural community integration (i.e., peer environment and following parental involvement in the community) has been compartmentalized.

Second, from the cross-sectional comparison, the current developmental space for urban children is composed of a school life environment, an out-of-school quality education environment, a recreational facilities environment (slides in neighborhoods, etc.), a virtual environment, and a parental companionship environment. In rural communities, there is basically no out-of-school quality education environment, suggesting that urban parents shape the companionship environment better than rural parents (Zhang, 2019).

The above longitudinal and cross-sectional comparisons show that the virtual and personalized content of rural children’s activities is the inevitable result of their monotonous developmental environment and their distance from the community environment.

### Meaning and Dilemmas of Community Inclusion for Rural Children

In the process of grounded theory construction, this study has experienced a shift from a focus on the developmental status of individual children to an examination of the developmental environment of children. In the selective coding, the theme of rural children community inclusion was gradually established. In recent decades, the dual cultural structure between urban and rural areas has been transformed in the process of urbanization. Because of its relatively backward mode of production and economic development level, rural culture lags behind urban culture in terms of the overall cultural development level. Therefore, rural culture has been in a disadvantaged position in the context of urban-rural integrated development. The parents of the farmers interviewed in this study generally did not want their children to be farmers. They showed a low sense of identification with farmers, which is a reflection of the disadvantaged position of rural culture. In that way, is it still necessary for rural children to include rural communities and identify with rural culture? This study will analyze this issue at the following two levels. First, the connotation of urban-rural culture integrated development has been developed in the background of the modernization of the national governance system and governance capacity (Yao, 2015). The Strategic Plan for Rural Revitalization of China (2018-2022) put forward the general requirement of “prosperous industry, ecological livability, civilized country, effective governance, and affluent living.” To achieve this ambitious goal, we must rely on current farmers and new farmers in the future. Therefore, the first tasks to accomplish rural revitalization are attaching enough importance to the community inclusion of rural children and building the cultural confidence of farmers. Second, the process of individual development involves continuous cultural adaptation. The core of urbanization is the urbanization of people. Individuals who grow up in rural areas and settle in cities will all experience bicultural identity integration. Existing research has found that migrant children with high bicultural identity integration exhibit better adaptive behavior (Li et al., 2018). Therefore, regardless of whether they live in rural or urban areas, rural children who complete their original cultural identity before adolescence will more easily integrate and adapt to society in the future.

### Reflections and Suggestions for Improving the Developmental Environment of Rural Children

This study found that parents widely supported rebuilding children’s community peer environment and affirmed the positive significance of traditional group play, which is consistent with the developmental niche theory (Super and Harkness, 1986, 1997). Social and play settings in daily routines can be an effective method for creating an interactive micro-environment between child and peers and parents, which is helpful for the learning and psychological development of the child. Besides, caretakers’ rearing beliefs and effective techniques contribute to the cultivation of children’s social, emotional, and cognitive rules of the traditional rural culture. Through in-depth interviews, parents put forward many constructive suggestions for rebuilding children’s community group games, which constitute conditions for the reconstruction of rural children’s peer environment. Therefore, based on the developmental niche theory and parents’ constructive opinions, this study offers the following reflections and suggestions for assisting children in achieving community inclusion and optimizing the environment for early childhood development.

#### Empowerment and Exploitation of Educational Functions in Rural Communities

Current rural early childhood development promotion programs are mostly implemented by outsiders with backgrounds in psychology, education, or social work and are mostly conducted through follow-up visits to households, which can lead to decontextualized and individualized interventions.

Context minimization error theory argued that psychological theories and research findings have ecological validity flaws and are only true in limited settings (Shinn and Toohey, 2003). Their research also suggested that the failure of many intervention treatments and community programs stems from the failure of intervention actors to understand the living environment of the intervention object (including family, friendships, networks, peer groups, neighbors, workplaces, schools, religious or community organizations, living areas, cultural heritage and cultural norms, gender roles, social and economic forces, etc.). For example, our project carried out family picture book reading empowerment in the pilot areas, using online punch cards for parents. On the one hand, this activity can help parents develop the habit of reading together with their children. On the other hand, it can achieve the effect of parents following each other’s successful experiences. In addition, research shows that parents can play the same role of education, guidance and support as teachers in the play engagement and game interaction with children in daily home context (Lin and Li, 2018). In the interviews for this study, we examined the attitudes and conditions of parental participation in parenting support groups, finding that the existing neighborhood and trust in rural communities could be used to rebuild children’s peer environments. Moreover, traditional group play is also an important cultural heritage in rural areas and plays a decisive role in rebuilding rural peer environments. Therefore, future intervention programs should focus on the development and use of this cultural heritage. The intervention actors ought to develop the existing educational resources in rural areas based on local perspectives to ensure the self-organization and self-running capacity of intervention programs.

#### Sustainable Development Pattern of “Relying on Education to Eliminate Poverty” Formed by Home-School-Community Cooperation

In rural areas, teachers are a group with relatively high cultural literacy who can not only take up the responsibility of teaching and educating but also drive the local cultural construction. The Strategic Plan for Rural Revitalization of China (2018-2022) specifically emphasized “carrying out activities such as searching for the most beautiful rural teachers” in the section on the prosperous development of rural culture. In the work of poverty elimination and rural style construction, teachers in certain areas have achieved good results by mobilizing students to urge parents to actively participate in rural construction, which also reflects the vital role of teachers in rural culture construction.

Therefore, family school partnerships should not be limited to schools and families working together to supervise children’s learning but rather to establish a large pattern of cooperation among schools, families, and communities. In this survey, we found that rural parents want to have professional teachers to lead parenting support groups. Additionally, the education bureau of the project demonstration area has been vigorously promoting the family school partnerships project, selecting excellent teachers from the teaching system for training, and eventually forming a team that can carry out family education work. Therefore, it is recommended that other poor areas should give full play to the role of teachers in building regional culture, especially training local excellent teachers in psycho-education and social work, so those rural areas can truly embark on a sustainable development path of “relying on education to get rid of poverty.”

### Limitations and Future Prospects

Although this article makes important contributions to systematic theoretical construction on the village inclusion of rural children, it has some potential limitations. First, our interview data were subjective self-reports based on qualitative research. Although this study has achieved good validity, it would be best to take quantitative research in the future to further test and verify the theoretical framework. Second, the interviewees in this study were mainly focused on the child’s mother. That is because female guardians (e.g., mothers), as the primary caregivers of children in the native culture, have a truer understanding of the development of their children. Nonetheless, we can’t ignore other key stakeholders’ views either. In the future, it may be considered to select children, male guardians, teachers, and village leaders for interviews to receive different perspectives on causes and effects of children’s rural inclusion, especially their attitudes toward socialization online. Third, despite the research sampling has reached theoretical saturation because there were not any new dimensions and key points emerging after the 17th respondent. It would be better to interview additional respondents to ensure that all the codes tended to be stable. Lastly, some interview questions have the guidance of value judgment, e.g., “what is your attitude about recovering traditional group games?” It would be better to change it to “what do you think of the future development trend of traditional group games?” It is suggested that the design of interview questions need to be more objective and neutral.

## Conclusion

The content and format of rural children’s activities showed increased participation in the virtual environment and weakened participation in the real community environment. In other words, rural children’s activities became more virtualized and personalized. In addition, rural parents and community peers are important bridges for children’s community inclusion. However, both the rural community peer environment and parental community participation showed a weakening tendency, which indicates that the two major channels for the community inclusion of rural children are blocked. This is also an important reason for the virtualization and individualization of children’s psychological developmental environment. Therefore, developmental intervention programs for rural children in poor areas should focus more on the reconstruction of children’s community peer environment. Furthermore, team managers should make efforts to encourage parents’ community participation to fully mobilize local culture-based educational resources.

## Data Availability Statement

The original contributions presented in the study are included in the article/Supplementary Material, further inquiries can be directed to the corresponding author/s.

## Ethics Statement

The studies involving human participants were reviewed and approved by the Ethics Committee for Scientific Research of Institute of Psychology, Chinese Academy of Sciences. The patients/participants provided their written informed consent to participate in this study. Written informed consent was obtained from the individual(s) for the publication of any potentially identifiable images or data included in this article.

## Author Contributions

LW was responsible for the research idea and study design. WY and YC provided efforts on collection of interview data and the first round of coding and analysis. YX and LW wrote the first version of this manuscript. WG, TT, and CF separately revised the manuscript and developed it. All authors contributed to the article and approved the submitted version.

## Conflict of Interest

The authors declare that the research was conducted in the absence of any commercial or financial relationships that could be construed as a potential conflict of interest.

## Publisher’s Note

All claims expressed in this article are solely those of the authors and do not necessarily represent those of their affiliated organizations, or those of the publisher, the editors and the reviewers. Any product that may be evaluated in this article, or claim that may be made by its manufacturer, is not guaranteed or endorsed by the publisher.
