# Supplementary material for: Problem Mechanism and Solution Strategy of Rural Children’s Community Inclusion—The Role of Peer Environment and Parental Community Participation
Source: Front Psychol. 2022 Jan 24;12:772362. doi: 10.3389/fpsyg.2021.772362 (PMC8820394; doi:10.3389/fpsyg.2021.772362)
Supplement: Supplementary file 1 [file Table_1.DOCX]

Supplementary Material

# Supplementary Figures and Tables

## Supplementary Tables

**Table 1.** Open coding and categorization of children’s psychological development environment

| **Categories** | **Examples of source sentence** | **Initial concept** | **Number**  **of** **node**  **samples** |
| --- | --- | --- | --- |
| External causes of virtual environment participation | N10 But it is not good for children's eyes and health after long-time phone use. | The disadvantages of mobile phone use | 6 |
|  | N4 He had no choice but to play poker and didn't read books until the evening. I was worried about him using the mobile phone, so I'd play poker with him for a while. | Play poker instead of mobile phone use | 1 |
|  | N7 Anyway, our families all swipe our phones. | Families play on mobile phones together. | 3 |
|  | N7 They each had a mobile phone to play on, and then I watched TV, thinking that one person had one phone, avoiding fights. | No restrictions on mobile phone use | 2 |
|  | C15 It is basically not available in our family that children play on mobile phones. We don't allow our kids to play on the phones. | Restrictions on mobile phone use | 8 |
|  | N8 She won't listen to what I say, yet she listens to her dad. If her dad allows her to watch, she does, and if he doesn’t agree, she doesn’t. | Difficult to restrict mobile phone use | 2 |
| Internal causes of virtual environment participation | N6 She just wants to find someone to play with every day, which makes me worry. She wants to find someone to play with and leads her to shop or go out on the street. When she sees a girl who is older than her, she runs to the front of the girl and grabs the girl’s hands, asking if she can play with her. | Child’s need for companionship | 2 |
|  | N13 I think the reason is that we parents usually play on the phone together. The most important thing is to spend less time on the phone and then accompany our kids. Although now the children are given the best to eat, drink, and play, but they have an extreme lack of company. | Lack of companionship | 1 |
|  | N2 She chooses her own cell phone, and she knows it better than her father. | Children's mobile phone use | 12 |
|  | N5 He watches TV at home every day. | Watch TV | 1 |
|  | N4 Three children can spend half a day on the phones when they sit together, and play their respective mobile games. | Substitution of playing on the phone for group games | 13 |
|  | N9 She wants to play (mobile phone) all day! | Long-term use of mobile phones | 4 |
| The function of peer games | N11 At that time, the children all played together, united with each other, and had a sense of teamwork. | Promote the spirit of cooperation | 5 |
|  | C14, On the other hand, I think outdoor activities are much better. | Do exercise | 3 |
|  | N12 Developing intelligence is useful. | Develop intelligence | 2 |
|  | N13 It is very important to study together, play games, and interact with each other, and learn to share with each other. | Learn to share | 1 |
|  | N9 What should I say about this? Yes. This should be attention and concentration. | Enhance attention | 1 |
|  | N10 It is like playing together and killing time. | Kill time | 3 |
|  | N9 I have feelings for my village, and these feelings come from my childhood friend, when I go home every Spring Festival. | Enhance the sense of community | 3 |
|  | N9 It can also improve the mood. | Enhance positive emotions | 8 |
|  | N9 The flip rope is also quite good, which is good for his imagination. | Enhance imagination | 2 |
| The attitude of peer environment reconstruction | N4 I want every child to come (to the library) on Saturdays and Sundays. I don't want them to be at home either. That's the state they're in at home (playing on their phones). So I'm going to make time for all the kids to come (to the library) every Saturday or Sunday. | Group game recovery support | 14 |
|  | N12 I feel it is very difficult (to recover). | Group games are difficult to recover | 1 |
|  | N13 I think it would be possible to gather the children and play games together. | Group games can be restored | 4 |
| Weakening of peer environment | N6 We just can't find children in this region. There are very few children at home. | There are few children in the village | 4 |
|  | N6 The key is older (children) don’t like to play with him. | The disappearance of the tradition of older children taking younger children to play | 1 |
|  | N5 Maybe because contemporary society is developed, things to play are more advanced. When we were children, in addition to playing this, there is no such thing as children playing now. | Play with toys alone | 4 |
|  | N5 There is one child in a family, and she does not play with others. Her own play interests are at home. For example, sometimes she puts on new clothes and enjoys them for a while, dances by herself, sometimes sings and watches TV. | Outdoor activities are reduced | 3 |
|  | N11 Nowadays, children do not have something to play with even on vacation, unlike when we were young with many playmates. | Community group games disappear | 14 |
|  | N13 Some events such as dances organized by the school were also cancelled. | School group games are weakened | 2 |
|  | N7 Now that the child is in third grade, he plays less. Because there is usually a lot of homework even on Saturday and Sunday. | Long working hours | 3 |
| Low sense of professional identity of parent | N12 My hope is to let her go to the military, nothing else is a big ideal. | Join the army | 1 |
|  | N5 I am a man who works in the fields, and I don't want my children to work in the fields in the future. | Don’t want children to work in the fields | 1 |
|  | N6 The future of farmers is not yet known. | The prospect of farmers is uncertain | 2 |
|  | N2 I hope she finds a rich husband. Daily life is to spend money, go to the mall, do not worry. How good it would be. | Marry a rich man | 1 |
|  | N13 I think many people will admire this kind of life, just like you have jobs. | Admire formal work | 3 |
|  | N7 (Farmers) don't earn much money and suffer exhaustion. | Low input-output ratio | 2 |
|  | N13 Actually, I have a lot of ideas. I hope he can become versatile, such as singing and dancing. I really want to cultivate him well to become all-round. Cause I like playing cello, dancing, and singing. | Writers and artists | 1 |
|  | N9 I hope that if he studies well, he may participate in work at least in the future. If not, he also needs a living skill of his own. | Have a skill | 1 |
|  | N7, I definitely want my kids to sit in the office. | Work in an office | 7 |
|  | N1 We are farmers, too, what's in it for us? Tired of working hard. | Being a farmer is very hard | 7 |
| Low sense of community of parent | N4 But when my grandparents came back, they both spent all day in Kuaishou (a short video software), and when they got older, they also watched Kuaishou with their mobile phones. | Parent mobile entertainment | 5 |
|  | N6 When I have time, but I'm afraid that others don't have time and delay their affairs. So I didn't visit. | Few visits with neighbors | 7 |
|  | N9 But my parents didn't care about me at that time. I would go out and run to play by myself. Now I think every family doesn't let children run to play by themselves. Adults are always behind children. | Low community trust | 5 |
| Obstacles to parenting support groups | N5 That is, grandparents look after my kid more. We seldom have time to teach him to read. | Early education is equivalent to children reading | 9 |
|  | N11 I’m worried to trust children to others! | Worried to trust children to others | 1 |
|  | N9 The difficulty is that some children don't listen to adults' stories at all. After all, rural children don't seem to like talking to strangers. I feel that it is difficult for children to talk with them. | Many children are afraid of strangers | 1 |
|  | N11 We can (take care of children) when we are free from farming. Once the farm is busy, you can't attend to it at all. | Lack of time to take care of children | 4 |
|  | C14, But I feel it may be difficult to maintain, so I'm afraid I can't keep it. | Hard to sustain | 1 |
|  | N12 It's hard to say when someone's child will bring something. Because children are especially spoiled now, if someone says there is a little bump or scratch, you can't make a difference. It's hard to say that if there will be any accidents when looking after other people's children. Because today's children are especially favored. Once a child is injured or scratched, it is difficult to explain to their parents. | Fear for others’ children getting hurt | 1 |
|  | Borrow (a book in the library)? Our family should take good care of him, or the books will all be torn up. | Fear for children tearing books | 2 |
|  | N13 There will definitely be contradictions. When a group of children get together, they will definitely grab things and fight, which is inevitable. | Fear of conflicts among children | 1 |
|  | N12 Unlike when we were kids being bumped, we just patted the soil and left. Nowadays, when children bump into each other, they need to be taken to a big hospital to check comprehensively. | Fear of caregiving responsibilities | 1 |
|  | C15 I think the younger children in this environment may not be able to sit still. Some children can't read, must need parents to speak to him, and when speaking may affect other children. | Concerns about group games | 3 |
|  | N12 I can't take care of those children by myself. Otherwise, the whole thing is out of order. Nowadays, children are very independent. | Inability to control multiple children | 1 |
| Conditions of parenting support groups | N11 Money does not always help. It's not good to pay. | Can't involve money | 1 |
|  | N10 I am also quite relieved. I am relieved with people I know well, the same as teachers. | Support attitude of parenting support groups | 8 |
|  | N12 The thing is that people communicate with each other. Familiar people are the same. It's okay for us to collide with familiar people. | Parents trust each other | 2 |
|  | N12 It's enough to have a rule in advance, otherwise it's abrupt and won't work. | Make rules | 1 |
|  | N13 I think parents have no ability to read and talk to them. They can recruit a teacher or somebody to tell them the content. | Professional teacher support | 2 |
| Benefits of parenting support groups | N12 Because my children are particularly afraid of meeting strangers. She never plays with strange children, but only plays with familiar children. | Exercise social skills | 1 |
|  | N12 It is fostering communication and unity among children. | Strengthen unity | 2 |
|  | N11 It's good that it was implemented. At least there is someone to babysit for me. | Reduce the pressure of nurture | 1 |
|  | N11 That's OK. I am able to look after two kids at the same time. | Improve the efficiency of looking after kids | 1 |
|  | N10 It is good for their studies to learn and play with each other. | Increase understanding | 2 |
|  | N13 In fact, if others babysit my child, they will pay more attention to him than their own children. | Enhance parental responsibility | 1 |
|  | N13 Bringing them together in this way will enhance their sense of unity. | Enhance the spirit of cooperation | 1 |
|  | N11 They play house together or something else. | Enhance group games | 1 |
